# Variability and Potential of Seaweeds as Ingredients of Ruminant Diets: An In Vitro Study

**DOI:** 10.3390/ani9100851

**Published:** 2019-10-22

**Authors:** Ana de la Moneda, Maria Dolores Carro, Martin R. Weisbjerg, Michael Y. Roleda, Vibeke Lind, Margarita Novoa-Garrido, Eduarda Molina-Alcaide

**Affiliations:** 1 Estación Experimental del Zaidin (Consejo Superior de Investigaciones Cientificas), Profesor Albareda, 1, 18008 Granada, Spain; anamonedarod@gmail.com; 2 Departamento de Producción Agraria. Escuela Técnica Superior de Ingeniería Agronómica, Alimentaria y de Biosistemas, Universidad Politécnica de Madrid, Ciudad Universitaria, 28040 Madrid, Spain; mariadolores.carro@upm.es; 3 Aarhus University, AU Foulum, Blichers Allé 20, 8830 Tjele, Denmark; martin.weisbjerg@anis.au.dk; 4 Norwegian Institute of Bioeconomy Research (NIBIO), PB 115, 1431 Ås, Norway; Michael.Roleda@nibio.no (M.Y.R.); vibeke.lind@nibio.no (V.L.); margarita.novoa-garrido@nord.no (M.N.-G.); 5 The Marine Science Institute, College of Science, University of the Philippines, Diliman, Quezon City 1101, Philippines; 6 Faculty of Biosciences and Aquaculture, Nord University, 8049 Bodø, Nordland, Norway

**Keywords:** seaweeds, chemical composition, in vitro rumen fermentation, goats, methane

## Abstract

**Simple Summary:**

The use of seaweeds as ingredients of ruminant diets can be an alternative to conventional feedstuffs, but it is necessary to assess their nutritive value. The aim of this study was to analyze the chemical composition and in vitro rumen fermentation of eight brown, red and green seaweed species collected in Norway during both spring and autumn. The in vitro ruminal fermentation characteristics of 17 diets composed of oat hay:concentrate in a 1:1 ratio, with the concentrate containing no seaweed or including one of the 16 seaweed samples, was also studied. Species and season determined differences in chemical composition and in vitro fermentation of seaweeds. Most of the tested seaweeds can be included in the diet (up to 200 g/kg concentrate) without negative effects on in vitro ruminal fermentation.

**Abstract:**

This study was designed to analyze the chemical composition and in vitro rumen fermentation of eight seaweed species (Brown: *Alaria esculenta*, *Laminaria digitata*, *Pelvetia canaliculata*, *Saccharina latissima*; Red: *Mastocarpus stellatus*, *Palmaria palmata* and *Porphyra* sp.; Green: *Cladophora rupestris*) collected in Norway during spring and autumn. Moreover, the in vitro ruminal fermentation of seventeen diets composed of 1:1 oat hay:concentrate, without (control diet) or including seaweeds was studied. The ash and N contents were greater (*p* < 0.001) in seaweeds collected during spring than in autumn, but autumn-seaweeds had greater total extractable polyphenols. Nitrogen in red and green seaweeds was greater than 2.20 and in brown seaweeds, it was lower than 1.92 g/kg DM. Degradability after 24 h of fermentation was greater in spring seaweeds than in autumn, with *Palmaria palmata* showing the greatest value and *Pelvetia canaliculata* the lowest. Seaweeds differed in their fermentation pattern, and autumn *Alaria esculenta*, *Laminaria digitata*, *Saccharina latissima* and *Palmaria palmata* were similar to high-starch feeds. The inclusion of seaweeds in the concentrate of a diet up to 200 g/kg concentrate produced only subtle effects on in vitro ruminal fermentation.

## 1. Introduction

The expected growth in the human population and the demand for animal products in the forthcoming years have increased the need for searching for alternative sources of nutrients for livestock feeding [1]. Seaweeds had been proposed as alternative feeds that might also have potential benefits on the health of the animals and the consumers of animal products due to their content in bioactive compounds [2,3]. Moreover, seaweeds offer additional advantages, as their cultivation does not compete with terrestrial agriculture, do not need fresh water, and the aquatic photosynthesis contribute to reduce CO_2_ levels. The use of seaweeds in animal feeding could also help to alleviate the environmental pollution caused by management of seaweeds in coastal zones. On the other hand, seaweed farming is known to render environmental benefits by recycling nutrients and preventing eutrophication [4].

Although there are studies [5] reporting the traditional use of seaweeds for feeding sheep in the Artic coastal areas and deers in Scotland and Alaska, their widespread use in ruminants is still limited, partly due to the lack of information on the species-specific variability in their the nutritional value and consistency in their chemical composition that may exhibit spatial (site-specific or regional) and temporal (i.e., seasonal and interannual) variations [6,7,8,9]. A characteristic common to all seaweeds is their high water content, which may be an important limitation to their direct use in livestock feeding. Another possible limitation is their high salt content [10]. In addition, the presence of compounds that can be a challenge for the digestive system of terrestrial animals may also limit the use of seaweeds in animal feeding [2]. Some recent studies have shown that seaweeds can contain bioactive compounds with antimetanogenic activity, and therefore, they could contribute to reducing the enteric CH_4_ emission from ruminants [11,12,13,14].

The use of seaweeds as ingredients of ruminant diets requires the assessment of their nutritive value. The first objective of this study was to investigate the chemical composition and in vitro ruminal fermentation of eight different species of seaweeds (three brown, four red and one green) harvested in Norway during spring and autumn. The second objective was to compare the in vitro ruminal fermentation of diets containing these seaweeds with a control diet not including seaweed that was formulated for goat feeding. The gas production technique was used for this study, as it is a relatively cheap and rapid technique that has being widely used in recent years for nutritive evaluation of different ruminant feeds, including seaweeds [8].

## 2. Materials and Methods

### 2.1. Seaweeds

The seaweeds used in the present study were chosen based on their biomass availability, potential for cultivation and traditional use for feeding livestock in the Artic areas where they were collected [15]. Eight different seaweed species were collected manually both in spring (March–April) and autumn (October–November) of 2015 in Bodø (northern Norway, 67°19′00″ N, 14°28′60″ E) during low tide. The tested seaweed species corresponded to three groups (Phyla) of seaweeds: the brown (Ochrophyta: *Alaria esculenta*, *Laminaria digitata*, *Pelvetia canaliculata* and *Saccharina latissima*), the red (Rhodophyta*: Mastocarpus stellatus, Palmaria palmata and Porphyra* sp.), and the green (Chlorophyta: *Cladophora rupestris*). The collected biomass was cleaned in a seawater bath to remove the remains of sand and associated fauna. Then, they were washed with a 30:70 mixture of seawater:freshwater, and finally, in fresh water to reduce the surface salt. The excess of surface water was manually drained and the seaweeds were frozen at −20 °C until their subsequent lyophilization. Once lyophilized, they were ground through a 1 mm sieve in a ZM 200 mill (Retsch GmbH, Haan, Germany).

### 2.2. Experimental Diets

Seventeen diets based on oat hay and concentrate in a 1:1 ratio were studied. The concentrate in the control diet was high in cereals (633 g/kg fresh matter) to be representative of those fed to goats in the practice and did not include any seaweed. Concentrates in the other 16 experimental diets included seaweeds (Table 1) replacing different amounts of feed ingredients (corn, wheat, soyabean meal, sunflower meal, palm soap and salts) present in the control concentrate. 

### 2.3. Donor Animals and Feeding

Four rumen-cannulated Murciano-Granadina goats with an average body weight of 43.8 ± 3.95 kg were used as donors of ruminal content for the in vitro incubations. The animals were fed a diet composed of oat hay and a commercial concentrate in a 50:50 ratio and were housed in pens in pairs with free access to drinking water. The level of intake was that of energy maintenance requirements [16] and the diet was supplied twice a day in equal amounts. The care and handling of the goats were carried out by trained personnel in accordance with the Spanish guidelines for the protection of animals used for experimentation or other purposes, and the experimental procedures were approved by the Animal Welfare Committee at the Zaidín Experimental Station of the Spanish National Research Council (Approval number: 05/24/2016/091).

### 2.4. In Vitro Trials

In vitro incubations were conducted using the seaweed samples alone and the 17 experimental diets (oat hay and concentrate 1:1) as substrates. Incubations were carried out in batch cultures of ruminal microorganisms using 120-mL glass bottles and ruminal fluid from goats as inoculum. The ruminal content was obtained from each of the four goats before the morning feeding, mixed, and immediately transported to the laboratory in thermal flasks pre-warmed at 39 °C. The ruminal content was filtered through four layers of surgical gauze and mixed with a buffer solution in a 1:4 ratio [17]; no trypticase added and under a continuous CO_2_ flow. A total of six incubation runs were carried out. In the first three incubation runs, seaweeds were used as substrate and three feeds commonly used in goat feeding (oat hay, barley straw and a commercial concentrate) were also included for comparative purposes. In the last three incubation runs, the substrates were the 17 experimental diets. In all the incubation runs, four bottles per substrate were used, and four blanks (bottles without substrate) were included.

Five hundred mg of each substrate were carefully weighed in each bottle and 60 mL of the mixture of ruminal fluid and buffer solution were added under a continuous flow of CO_2_. Bottles were sealed with butyl rubber stoppers and aluminum caps and placed in a water bath at 39 °C. The pressure inside the bottles and the volume of gas produced in two bottles per substrate and two blanks were measured at 2, 4, 6, 8, 12, 24, 48, 72, 96, 120 and 144 h of incubation using a pressure gauge scope (Sper Scientific LTD, Scottsdale, AZ, USA) and a calibrated glass syringe (Ruthe^®^, Normax Marinha Grande, Portugal). Additionally, in the incubations using seaweeds as substrate, the content of each bottle at the end of the 144 h of incubation was weighed, frozen at −20 °C and analyzed for neutral detergent fibre (NDF) content to estimate the true dry matter (DM) digestibility (TDMD_144_) as described by Van Soest et al. [18].

In each incubation run, the other two bottles for each substrate (either seaweeds or the experimental diets) and blanks were incubated for 24 h. Gas production measurement was done as described above and a gas sample (5 mL) was stored in a vacuum tube (Terumo Europe NV, Leuven, Belgium) for analysis of CH_4_. The fermentation was stopped by chilling on ice. The content of the bottles was homogenized and the following samples were taken: 2 mL were added to 2 mL of a deproteinizing solution (20 g of metaphosphoric acid and 0.6 g of crotonic acid per liter) for the analysis of volatile fatty acids (VFA), and 1 mL was mixed with 1 mL of 0.5 M HCl for the analysis of NH_3_-N. Additionally, in the incubations using seaweeds as substrate, the content of each bottle was weighed (before sampling), frozen at −20 °C, and analyzed for neutral detergent fibre (NDF) content to estimate the true dry matter (DM) digestibility (TDMD_24_) as described by Van Soest et al. [18].

### 2.5. Chemical Analyses

The DM content of the seaweeds and experimental concentrates was determined by lyophilization and subsequent drying of the lyophilized material in an oven at 103 °C for 24 h [19]. Ash content in seaweed (ID 048.13) and ether extract (ID 945.16) were determined according the AOAC procedures [19]. The total N content was analyzed by the Kjeldahl method. The NDF content in the in vitro incubation residues was determined following the procedure of Goering and van Soest [17] using a FibertecTMM6 system (Foss Analytical, Hillerød, Denmark). In the NDF analysis of concentrates, heat-stable amylase was added [20], and all results are expressed as ash-free. The content in total extractable polyphenols (TEP) was analyzed following the procedure of Julkunen-Tiito [21]. The concentrations of individual VFA in the content of the bottles and CH_4_ in the gas produced were analyzed by gas chromatography using a HP Hewlett 5890 Packard Series II gas chromatograph (Waldbronn, Germany) equipped with a flame ionization detector (FID) and an HPINNOWAX cross linked polyethylene glycol column (25 m× 0.2 mm× 0.2 μm; Teknokroma, Madrid, Spain) as described by Molina-Alcaide et al. [8]. The concentration of N-NH_3_ was determined following the colorimetric method of Weatherburn [22] using a spectrophotometer (Thermo Scientific, Genesys 10 uV Scanning, Madison, WI 53711 USA).

### 2.6. Calculations and Statistical Analysis

The gas production data were adjusted to the exponential model: gas = A (1 − e ^(−c (t-lag)^), where A is the asymptotic gas production, c is the gas production rate, lag is the delay at the start of gas production, and t is the time of gas measurement. Parameters A, c and lag were estimated using an iterative least-square procedure following the NLIN procedure of SAS (version 9.4, SAS Inst. Inc., Cary, NC, USA). The average gas production rate (AGPR, ml/h) is defined as the average rate of gas production between the start of incubation and the time at which half of A is reached, and was calculated as AGPR = A c/[2 (ln2 + c lag)]. The amount of VFA in each bottle after 24 h of incubation was corrected by the amount of VFA added with the ruminal fluid used as inoculum.

Data on the chemical composition of seaweed were analyzed by ANOVA using the PROC GLM of SAS (version 9.4, SAS Inst. Inc., Cary, NC, USA) in which the seaweed species and harvest season were the main effects. Fermentation data of seaweeds were analyzed using the PROC MIXED of SAS as a mixed model (version 9.4, SAS Inst. Inc., Cary, NC, USA), in which the seaweed species, harvest season and seaweed species x season interaction were considered as fixed effects, and the incubation run was considered random. The model for the analysis of data of experimental diets included the fixed effect of diet and the random effect of the incubation run. When a significant effect was detected (*p* ≤ 0.05), the differences between the means were tested using Tukey’s multiple comparison test.

## 3. Results

### 3.1. Chemical Composition and In Vitro Fermentation of Seaweeds

Both seaweed species and harvest season affected (*p* < 0.001) all chemical fractions analyzed (Table 2). Ash and N content was greater (*p* < 0.001) in seaweeds collected in spring than in those harvested in autumn (224 vs. 121 g/kg DM and 3.08 vs. 1.92 g/kg DM, respectively). Ash content ranged from 88.2 g/kg DM in *Porphyra* sp. to 225 g/kg DM in *Laminaria digitata* and *Saccharina latissima* (values averaged across seasons). There were also wide variations in total N content, with red and green seaweeds having values greater than 2.20 g/kg DM (values averaged for both collection seasons) and brown species showing values lower than 1.90 g/kg DM. The TEP content was greater (*p* < 0.001) in autumn than in spring (12.1 vs. 6.82 g/kg DM), and the greatest values corresponded to *Alaria esculenta* and *Pelvetia canaliculata*.

As shown in Table 3, seaweed species x season interactions (*p* < 0.001) were detected for all the parameters of gas production (A, *c*, *lag* and AGPR) and TDMD_244_. There were differences (*p* < 0.001) among seaweed species in all the parameters of gas production and in vitro digestibility values. *Palmaria palmata* had the greatest (*p* < 0.05) A and AGPR values (143 mL and 4.95 mL/g DM, respectively) with A values being similar to those in the three feedstuffs used as reference and AGPR values higher than those for feedstuffs. The lowest (*p* < 0.05) values were shown by *Pelvetia canaliculata* (8.2 mL and 1.38 mL/g DM, respectively, for A and AGPR) and were much lower than A for any of the feedstuffs and AGPR similar to this value in barley straw. The *lag* values were 0.00 for most seaweed samples, with the exception of *Alaria esculenta* in autumn, *Saccharina latissima* in spring and *Palmaria palmata*, but all the values were lower than 1 h except those for *Alaria esculenta* in autumn (2.58 h). The collecting season affected (*p* < 0.001) the values of A, *lag*, AGPR and TDMD_24_. Compared with spring seaweeds, those collected in autumn had greater A (65.5 vs. 87.5 mL), lag (0.01 vs. 0.42 mL) and AGPR (2.14 vs. 2.93), but lower TDMD_144_ values (87.9 vs. 83.0%).

There were differences (*p* < 0.001 to 0.003) among seaweed species in total VFA production, VFA profile and acetate/propionate ratio (Table 4). *Pelvetia canaliculata* had the lowest (*p* < 0.05) VFA production, whereas *Alaria esculenta* and *Saccharina latissima* had the greatest production (*p* < 0.05). The VFA production was not affected (*p* = 0.821) by the harvesting season, and no seaweed species x season interaction (*p* = 0.609) was detected. In contrast, seaweed species x season interactions (*p* < 0.001) were detected for molar proportions of acetate, propionate, isobutyrate and isovalerate. *Palmaria palmata* had the lowest proportion of acetate and the greatest propionate proportion (58.5% and 30.1%, respectively), whereas *Mastocarpus stellatus* had the lowest proportion of propionate and the greatest of butyrate (15.1% and 9.50%). The production of minor VFA (isobutyrate, isovalerate and valerate) also differed among seaweed species, with *Porphyra* sp. having the greatest (*p* < 0.05) proportions of isobutyrate and isovalerate and *Pelvetia canaliculata* the greatest valerate proportions. Compared to seaweeds harvested in spring, autumn seaweeds had lower (*p* < 0.001 to 0.020) proportions of acetate (69.0% vs. 59.3%) and minor VFA, as well as greater propionate (18.4% vs. 27.1%) and butyrate (6.37% vs. 8.87%) proportions. The acetate/propionate ratio was highly variable, with values ranging from 1.47 mol/mol in *Alaria esculenta* to 4.76 mol/mol for *Mastocarpus stellatus* both collected in autumn. Spring seaweeds had greater (*p* < 0.001) acetate/propionate ratios than those collected in autumn (3.91 vs. 2.52).

Both species and season affected (*p* < 0.001) NH_3_-N concentrations, CH_4_ production and TDMD_24_, (Table 4), and seaweed species × season interactions were detected for NH_3_-N concentrations and TDMD_24_. *Alaria esculenta* and *Porphyra* sp. had the lowest and greatest NH_3_-N concentrations, respectively, whereas *Pelvetia canaliculata* and *Palmaria palmata* had the lowest and greatest CH_4_ productions, respectively. The values of TDMD_24_ ranged from 63.0% to 91.8%, the lowest and greatest values corresponding to *Cladophora rupestris* and *Palmaria palmata*, respectively. Greater (*p* < 0.001) NH_3_-N concentrations and TDMD_24_ values and lower (*p* < 0.001) CH_4_ production were observed for the samples collected in spring (17.8 mg/100 mL, 84.5% and 26.7 mL, respectively) compared to those collected in autumn (9.99 mg/100 mL, 78.1% and 33.4 mL).

### 3.2. Chemical Composition and In Vitro Fermentation of Experimental Diets

The chemical composition of the experimental diets is shown in Table 5. In general, ash content was greater in the diets containing seaweeds than in the control diet, whereas the opposite was observed for ether extract content. There were only small differences among diets in N content, which ranged from 17.6 to 19.8 g/kg DM, whereas TEP content varied from 4.75 to 7.98 g/kg DM.

As shown in Table 6, the diets including *Palmaria palmata* collected in autumn, and *Porphyra sp* and *Cladophora rupestris* collected in spring and autumn had greater (*p* < 0.05) potential gas production values (A) compared with the rest of the diets, including the control one. All the diets including seaweeds, except that with *Palmaria palmata* collected in autumn, had lower (*p* < 0.05) fractional rates of gas production and AGPR than the control.

Table 7 shows the in vitro fermentation parameters of the experimental diets. There were no differences (*p* ≥ 0.152) in total VFA production, minor VFA molar proportions and NH_3_-N concentrations. Compared with the control, diets including spring-harvested *Alaria esculenta*, *Saccharina latissima*, *Palmaria palmata*, *Laminaria digitata*, *Pelvetia canaliculata,* and *Mastocarpus stellatus* from both seasons had greater (*p* < 0.05) acetate proportions. All the diets except that including autumn-harvested *Alaria esculenta* had lowers (*p* < 0.05) propionate molar proportions than the control. Butyrate molar proportions were lowest for the diets with *Alaria esculenta, Laminaria digitata* and *Saccharina latissima* and greatest for the diets with *Porphyra sp.* and *Cladophora rupestris*, with the control diet having an intermediate value. Most diets including seaweeds had greater (*p* < 0.05) acetate/propionate ratios than the control diet, except those including autumn-harvested *Alaria esculenta*, *Laminaria digitata, Saccharina latissima* and *Palmaria palmata.* All the diets with autumn-harvested seaweeds had lower CH_4_ production than the control diet.

## 4. Discussion

### 4.1. Chemical Composition and In Vitro Fermentation of Seaweeds

The low DM and high ash content of seaweeds are frequently reported as the main limitations to their use in ruminant diets [7,8]. Both DM and ash contents were similar to those reported for the same seaweeds and others (*Ruppia maritima*, *Ulva lactuca* and *Chaetomorpha linum*) in previous studies [7,23]. In accordance with Tayyab et al. [7], the ash content of seaweeds was greater in spring than in autumn, and the values were greater than those found in conventional feeds used in ruminant nutrition (Table 2). As previously reported [7,8,24,25]. The N content was highly variable, and it was greater in spring-harvested seaweeds than in those collected in autumn. This has been attributed to high sunlight conditions that increase the photosynthesis and nutrient assimilation and to greater N concentration in water during spring compared with autumn [24]. Both *Porphyra sp* and *Cladophora rupestris* showed an N content greater than that in the commercial concentrate used as reference in our study (Table 2; 23.0 g/kg DM), but other seaweeds had an N content similar to that in the oat hay or even lower, especially those harvested in autumn. High-protein seaweeds may be used as an alternative to conventional high-protein feeds, such as soybean meal, and recent studies [25] showed that some amino acids in *Laminaria* and *Mastocarpus* species were protected against rumen degradation, making them potential sources of by-pass protein. In agreement with previous studies [8,9,26], brown seaweeds had, in general, a greater TEP content than both red and green seaweeds, and TEP content was lower in spring-harvested seaweeds than in those collected in autumn. Brown seaweeds are rich in phlorotannins [27], which seem to be different from the tannins in terrestrial plants, but their effect on ruminants is still unknown. Polyphenols have been reported to reduce protein degradation in the rumen, but they can also reduce the fibre degradation by decreasing the attachment of microbes to feed particles [3]. The negative relationships (n = 16) observed between the TEP content and TDMD_144_ (r = 0.732; *p* = 0.001), TDMD_24_ (r = 0.503; *p* = 0.047), and total VFA concentrations (r = 0.478; *p* = 0.061) indicates a negative effect of TEP on the in vitro rumen degradation of seaweeds. However, there were no correlations between TEP content and any of the gas production parameters, which supports the idea that gas measurement should be combined with measurements of feed degradability for a better interpretation of polyphenols effects, as pointed out by Makkar [3].

The high variability observed in the potential gas production values (A) of seaweeds reflects the differences in their potential degradation in the rumen. In fact, a positive relationship between A and TDMD_144_ (r = 0.510; *p* = 0.044; n = 16) was detected. The lowest A and TDMD_144_ values were observed for *Pelvetia canaliculata*, which agrees with the low DM degradability values reported for this seaweed by Tayyab et al. [7] using the in situ technique in dairy cows and by Molina-Alcaide et al. [8] in 24-h in vitro incubations with sheep ruminal fluid. The greatest A and TDMD_144_ values were observed for *Palmaria palmata* and *Saccharina latissima*, which is in agreement with the high ruminal degradability observed in previous studies for both seaweeds [7,8].

A 24-h incubation period was chosen for the in vitro incubations in our study, as this rumen retention time can be found in goats and sheep fed at moderate levels of intake [28,29]. In agreement with the results of the gas production study, *Pelvetia canaliculata* promoted the lowest total VFA production, which was only 0.41 of that observed for barley straw, and *Palmaria palmata* and *Saccharina latissima* had the greatest values, which were 1.3 and 0.75 of those observed for the concentrate, respectively. Total VFA production for *Porphyra sp.* and *Cladophora rupestris* was similar to that for barley straw, whereas the fermentation of *Alaria esculenta* and *Laminaria digitata* promoted a VFA production only slightly lower than that from fermentation of medium-quality forage such as the oat hay used in our study. These results show that seaweeds can be fermented in the rumen to a variable extent. Although the collecting season had a marked influence on the chemical composition of seaweeds, no differences between seasons were observed in total VFA production. This agrees with the lack of differences between the two harvesting seasons in the ruminal degradability of the protein of nine seaweed species observed by Gaillard et al. [25], despite the marked differences detected in protein content. 

There were pronounced differences among seaweed species with regard to VFA profile. *Alaria esculenta, Laminaria digitata, Saccharina latissima* and *Palmaria palmata* harvested in autumn had high propionate proportions (≥32.7%) and their acetate/propionate ratio (1.47:1.70) was similar to that observed in ruminants fed diets based on high-cereal concentrates [30,31]. Conversely, seaweeds harvested in spring, except *Palmaria palmata*, had acetate/propionate ratios (3.13:4.73) similar or even greater than those observed for the oat hay and barley straw used as reference, and the values were similar to those reported in forage-fed ruminants [32,33,34]. High variations between seaweed species in the in vitro VFA profile have also been previously observed [8,13,14].

The degradation of some amino acids produces branched-chain VFA, and therefore, they can be used as an index of protein degradation [35]. *Cladophora rupestris* and *Porphyra sp.* had the greatest N content (37.1 and 55.4 g/kg DM, respectively) and also the greatest proportions of minor VFA (calculated as the sum of isobyutyrate, isovalerate and valerate; 9.55% and 7.83%), whereas *Alaria esculenta* had the lowest proportions of minor VFA (2.47%) despite having an intermediate N content (18.2 g/kg DM). As pointed out by Hume [36], the interpretation of isoacids proportions is difficult because they are captured and used by the cellulolytic bacteria and the analyzed concentrations are the balance between the N produced from degradation and the N used by the bacteria to synthesize microbial protein in the rumen. Despite this, in our study the proportions of minor VFA were positively correlated with the N content of seaweeds (r = 0.730; *p* = 0.001; n = 16).The N content was also positively correlated with NH_3_-N concentrations (r = 0.952; *p* < 0.001; n = 16), which reflects the balance between the NH_3_-N produced by protein degradation and that captured by ruminal microorganisms. The NH_3_-N concentrations for most of the seaweeds were above the level limiting in vitro ruminal microbial growth (5 mg/100 mL) [37], but concentrations for autumn-harvested *Alaria esculenta, Laminaria digitata, Saccharina latissima and Palmaria palmata* were clearly below this level (≤ 1.58 mg/100 mL), suggesting a possible limitation of microbial growth. These seaweeds had both low N content (ranging from 6.03 g/kg DM in *Saccharina latissima* to 14.6 g/kg DM in *Palmaria palmata*) and low proportions of minor VFA (1.20 in *Alaria esculenta* to 2.72% in *Saccharina latissima*), which would indicate low protein degradation. Interestingly, these seaweed samples promoted a high-propionate fermentation pattern (≥32.7% propionate), suggesting that the low NH_3_-N concentrations could also have been due to a high NH_3_-N capture by ruminal microorganisms, as was reported to occur in ruminants fed diets based on high-cereal concentrates [31,38].

The production of CH_4_ from seaweed fermentation was highly variable, but the positive correlation observed between CH_4_ and total VFA production (r = 0.881; *p* < 0.001; n = 16) suggests that the observed differences can be partly explained by the amount of substrate fermented, as both VFA and CH_4_ derive from organic matter fermentation [8]. Several studies have investigated the possible antimethanogenic effect of marine seaweeds, with controversial results. Belanche et al. [12] observed no changes in in vitro CH_4_ emissions when *Laminaria digitata* or *Ascophyllum nodosum* were included in the diet at 50 g/kg DM. However, Kinley et al. [14] and Machado et al. [39] observed an antimethanogenic effect of *Asparagopsis taxiformis* included in the diet at 20 g/kg, and Machado et al. [39] observed similar effects for a freshwater/brackish alga *Oedogonium* sp. at greater doses (>500 g/kg). The CH_4_/total VFA ratio in the seaweeds (Table 4) was similar or slightly lower than that of the concentrate used as reference (10.3 mL/mmol), except for *Pelvetia canaliculata* (5.05 mL/mmol), *Porphyra sp.* (11.7 mL/mmol) and *Cladophora rupestris* (12.6 mL/mmol). The greater CH_4_/VFA ratio observed in *Porphyra* sp. and *Cladophora rupestris* might be related to their high N content, as it has been shown that protein fermentation also contributes to CH_4_ formation [40].

### 4.2. Chemical Composition and In Vitro Fermentation of Experimental Diets

The level of seaweed inclusion in the concentrates was chosen from its N content and degradability with the aim that all diets had a similar N content [7]. However, a maximum of 200 g of seaweed per kg concentrate was set up following the recommendations of Rjiba-Ktita et al. [23], who observed that inclusion levels of different seaweed species greater than 200 g/kg reduced the rate and extent of degradation of the mixture. In addition, a minimum of 93 g of soyabean meal per kg concentrate was fixed to guarantee the supply of essential amino acids (mainly lysine) for the host ruminant. *Alaria esculenta*, *Laminaria digitata*, *Pelvetia canaliculata* and *Saccharina latissima* were included as energy sources and therefore, they replaced different amounts of wheat bran, corn and wheat in the concentrate. *Mastocarpus stellatus* and *Palmaria palmata* were included as sources of both energy and protein, and therefore, they replaced different amounts of wheat bran, corn, soyabean meal and sunflower meal. Finally, *Porphyra* sp. and *Cladophora rupestris* were considered as protein sources and replaced both soyabean meal and sunflower meal.

The slightly lower N content observed in the diets including autumn-seaweed compared with those including spring-seaweed is consistent with the lower N content of the autumn seaweeds, as both spring and autumn samples of each seaweed were included in the same proportion in the diet (Table 1). The inclusion of seaweeds in the diet resulted in lower *c* and AGPR values than those in the control diet, which indicates that seaweeds were slower fermented than the conventional feeds (wheat, corn, soyabean meal, sunflower meal) they replaced in the concentrate. It has to be taken into account that the differences observed among diets in fermentation parameters are not only due to the inclusion of seaweeds, but also to the different proportions of each feed included in the corresponding concentrate. The diet including *Palmaria palmata* collected during autumn was the only seaweed that had *c* and AGPR values similar to those in the control diet, which was due to their rapid fermentation rate. As indicated by the values of the potential gas production (A), the inclusion of seaweeds in the concentrates at the level used in this study did not reduce the extent of fermentation, and in some cases (autumn-harvested *Palmaria palmata* and *Porphyra* sp. and *Cladophora* collected in both spring and autumn), even confirmed it.

The lack of negative effects of the seaweeds on the in vitro degradation of the diets was confirmed by the absence of differences among diets in total VFA production. In contrast, there were some differences among diets in the VFA profile, and acetate/propionate ratio was greater than that in the control diet for all seaweeds except *Alaria esculenta*, *Laminaria digitata*, *Saccharina latissima*, and *Palmaria palmata* collected in autumn. These results are in agreement with the low acetate/propionate ratios observed in the fermentation of these seaweeds, which was similar to those observed for ruminants fed high-cereal diets. The lack of differences among diets in NH_3_-N concentrations and minor VFA proportions is in accordance with the similar N contents in all the diets and also indicates similar protein degradability in all the diets.

There were some differences among diets in CH_4_ production, and the diets containing *Laminaria digitata* and *Mastocarpus stellatus* collected in autumn showed the lower values. The ratio CH_4_/VFA can be used as an indicator of the efficiency of ruminal fermentation, as CH_4_ is an energy loss to the host animal and VFA is used as an energy source and as substrates for the synthesis of other compounds [41]. The similar values of this ratio observed for all diets (*p* = 0.569) indicate that the observed differences in CH_4_ production were mostly due to the amount of substrate fermented. The positive correlation observed between CH_4_ and total VFA production (r = 0.816; *p* < 0.001; n = 17) supports this hypothesis. As discussed above, differences among diets in both CH_4_ and VFA production are not only due to the inclusion of seaweeds, but also to the different feed ingredients in the concentrate. These results indicate that none of the tested seaweeds had a noticeable antimethanogenic effect.

## 5. Conclusions

The composition of the seaweeds was variable depending on both species and the harvesting season, with seaweeds collected in autumn having less N and ash and more polyphenols than spring-harvested seaweeds. The brown seaweeds studied are sources of energy, whereas *Porphyra sp*. and *Cladophora rupestris* are good protein sources and can be used as substitutes for conventional protein feeds. Seaweeds differed in their ruminal fermentation pattern and autumn-harvested *Alaria esculenta*, *Laminaria digitata*, *Saccharina latissima* and *Palmaria palmata* were similar to conventional high-starch feeds used in ruminant feeding. The inclusion of variable levels of seaweeds in the concentrate of a diet (up to 200 g/kg concentrate) produced only subtle effects on in vitro ruminal fermentation.

## Figures and Tables

**Table 1:** Ingredient composition (g/kg fresh matter) of the experimental concentrates ^1^.

Ingredient	Control	AS	AA	LS	LA	PS	PA	SS	SA	MS	MA	PPS	PPA	POS	POA	CS	CA
Wheat bran	250	165	165	166	166	186	186	250	250	250	250	195	195	250	250	250	250
Corn	250	250	250	245	245	164	164	100	100	159	159	206	206	250	250	250	250
Wheat	133	18	18	22	22	133	133	133	133	133	133	133	133	133	133	133	133
Soybean meal	124	124	124	124	124	124	124	124	124	104	104	93	93	100	100	96	96
Sunflower meal	84	84	84	84	84	84	84	84	84	45	45	84	84	24	24	28	28
Soybean husk	100	100	100	100	100	100	100	100	100	100	100	100	100	100	100	100	100
Others ^2^	59	59	59	59	59	59	59	59	59	59	59	59	59	59	59	59	59
Seaweed	-	200	200	200	200	150	150	150	150	150	150	130	130	84	84	84	84

^1^ Each experimental concentrate contained one seaweed (A: Alaria esculenta; L: Laminaria digitata; P: Pelvetia canaliculata; S: Saccharina latissima; M: Mastocarpus stellatus; PP: Palmaria palmata; PO: Porphyra sp.; C: Cladophora rupestris harvested either in spring (AS, LS, PS, SS, MS, PPS, POS and CS concentrates) or in autumn (AA, LA, PA, SA, MA, PPA, POA and CA concentrates). ^2^ All the concentrates included: 10 g of calcium carbonate, 10 g of sugarbeet molasses, 10 g of sepiolite, 14 g of palm soap, 5 g of NaCl, 5 g of Na_2_CO_3_, and 5 g of mineral-vitamin mixture per kg.

**Table 2:** Chemical composition (g/kg dry matter unless otherwise stated) of different seaweed species harvested in spring and autumn in northern Norway and of feeds commonly used in ruminant diets.

Species	Season	Dry Matter	Ash	Nitrogen	Total Extractable Polyphenols
		(g/100 g Fresh Matter)			
Brown seaweeds					
*Alaria esculenta*	Spring	110	288	23.4	4.51
	Autumn	277	73.6	13.1	28.1
	Average	193 ^f^	181 ^d^	18.2 ^d^	16.3 ^e^
*Laminaria digitata*	Spring	115	311	23.0	1.44
	Autumn	189	138	6.77	6.08
	Average	152 ^b^	225 ^e^	14.9 ^c^	3.76 ^d^
*Pelvetia canaliculata*	Spring	237	199	16.3	26.9
	Autumn	237	174	6.88	40.4
	Average	237 ^g^	187 ^d^	11.6 ^a^	33.7 ^f^
*Saccharina latissima*	Spring	87.0	350	17.6	3.87
	Autumn	220	100	6.03	5.21
	Average	154 ^c^	225 ^e^	11.8 ^a^	4.54 ^d^
Red seaweeds					
*Mastocarpus stellatus*	Spring	261	183	26.4	4.36
	Autumn	245	194	18.1	3.57
	Average	253 ^h^	189 ^d^	22.2 ^e^	3.97 ^ab^
*Palmaria palmata*	Spring	121	213	43.0	3.86
	Autumn	191	103	14.6	1.93
	Average	156 ^d^	158 ^c^	28.8 ^f^	2.89 ^b^
*Porphyra sp.*	Spring	90.0	97.9	59.8	4.75
	Autumn	116	78.4	50.9	5.85
	Average	103 ^a^	88.2 ^a^	55.4 ^h^	5.30 ^c^
Green seaweeds					
*Cladophora rupestris*	Spring	191	149	37.1	4.88
	Autumn	181	105	37.0	5.39
	Average	186 ^e^	127 ^b^	37.1 ^g^	5.14 ^bc^
*p* value					
Species		<0.001	<0.001	<0.001	<0.001
Season		<0.001	<0.001	<0.001	<0.001
SEM		0.004	0.670	0.128	0.035
Feeds					
Oat hay		896	62.7	12.7	6.82
Barley straw		941	43.7	3.07	NA ^1^
Commercial concentrate		933	77.4	23.0	NA ^1^

^a–e^ For each parameter, average values for each seaweed not sharing the same superscript differ (*p* < 0.001); ^1^ NA: not analysed.

**Table 3:** Parameters of gas production kinetics (A, c, *lag* and AGPR) and true dry matter (DM) digestibility (TDMD_144_) after 144 h of in vitro incubation of different seaweed species harvested in spring and autumn in northern Norway and of feeds commonly used in ruminant diets ^1^.

Seaweed Species	Season	A (ml)	*c* (h^−1^)	*lag* (h)	AGPR (ml/h)	TDMD_144_ (%)
Brown seaweeds						
*Alaria esculenta*	Spring	85.9	0.034	0.00	2.11	93.2
	Autumn	104.9	0.033	2.58	2.20	75.4
	Average	95.4 ^e^	0.034 ^a^	1.29 ^c^	2.16 ^b^	84.3 ^c^
*Laminaria digitata*	Spring	85.2	0.027	0.00	1.68	98.3
	Autumn	107.4	0.034	0.00	2.59	79.1
	Average	96.3 ^e^	0.031 ^a^	0.00 ^a^	2.14 ^b^	88.7 ^d^
*Pelvetia canaliculata*	Spring	6.3	0.351	0.00	1.58	67.8
	Autumn	10.0	0.162	0.00	1.17	68.4
	Average	8.15 ^a^	0.257 ^b^	0.00 ^a^	1.38 ^a^	68.1 ^a^
*Saccharina latissima*	Spring	84.0	0.030	0.07	1.82	97.6
	Autumn	147.1	0.043	0.00	4.58	94.6
	Average	116 ^f^	0.037 ^a^	0.04 ^a^	3.20 ^c^	96.1 ^e^
Red seaweeds						
*Mastocarpus stellatus*	Spring	31.0	0.068	0.00	1.52	89.3
	Autumn	20.6	0.078	0.00	1.16	91.0
	Average	25.8 ^b^	0.073 ^a^	0.00 ^a^	1.34 ^a^	90.2 ^d^
*Palmaria palmata*	Spring	114.6	0.060	0.03	4.93	95.8
	Autumn	171.9	0.042	0.74	4.97	96.4
	Average	143 ^g^	0.051 ^a^	0.39 ^b^	4.95 ^d^	96.1 ^e^
*Porphyra sp.*	Spring	54.8	0.063	0.00	2.51	87.3
	Autumn	64.7	0.071	0.00	3.31	90.0
	Average	59.8 ^c^	0.067 ^a^	0.00 ^a^	2.91 ^c^	88.7 ^d^
Green seaweeds						
*Cladophora rupestris*	Spring	62.4	0.020	0.00	0.99	73.5
	Autumn	73.1	0.066	0.00	3.47	74.3
	Average	67.8 ^d^	0.043 ^a^	0.00 ^a^	2.19 ^b^	73.9 ^b^
						
*p value*						
Species		<0.001	<0.001	<0.001	<0.001	<0.001
Season		<0.001	0.301	<0.001	<0.001	<0.001
Species x season		<0.001	<0.001	<0.001	<0.001	<0.001
SEM		0.296	0.008	0.029	0.057	0.347
						
Feeds						
Oat hay		129.1	0.037	0.00	3.43	79.7
Barley straw		124.6	0.017	0.41	1.53	56.9
Commercial concentrate		146.3	0.064	0.00	6.75	91.4

^a–e^ For each parameter, the average values for each seaweed not sharing the same superscript differ (*P* < 0.05). ^1^ A: asymptotic gas production; *c:* rate of gas production; lag: lag time before fermentation starts; AGPR: average gas production rate; DMED_24_: dry matter effective degradability calculated for a rumen passage rate of 0.041 per h. Data are expressed per 0.5 g DM fermented.

**Table 4:** Fermentation parameters and true dry matter (DM) digestibility (TDMD_24_) after 24 h of in vitro incubation of different seaweed species harvested in spring and autumn in northern Norway and of feeds commonly used in ruminant diets.

Seaweed Species	Season	VFA (mmol/g DM)	Molar Proportions (mol/100 mol)						Acetate/Propionate (mol/mol)	NH_3_-N (mg/100 mL)	CH_4_ (mL/g DM)	CH_4_/VFA (mL/mmol)	TDMD_24_ (%)
			Acetate	Propionate	Butyrate	Isobutyrate	Isovalerate	Valerate					
**Brown Seaweeds**													
*Alaria esculenta*	Spring	3.44	71.1	19.3	5.80	0.78	1.09	1.86	3.69	7.52	28.6	8.31	90.8
	Autumn	3.42	53.6	37.1	8.18	0.02	0.03	1.15	1.47	1.58	28.7	8.39	75.5
	Average	3.43 ^cd^	62.4 ^b^	28.2 ^de^	7.0 ^ab^	0.40 ^a^	0.56 ^a^	1.51 ^a^	2.58 ^b^	4.55 ^a^	28.7 ^b^	8.37 ^b^	83.2 ^c^
*Laminaria digitata*	Spring	3.16	76.1	16.2	4.00	0.80	1.17	1.75	4.73	10.7	17.0	5.38	92.6
	Autumn	3.24	54.8	32.7	10.0	0.66	0.50	1.32	1.70	0.80	38.5	11.9	75.7
	Average	3.20 ^b c^	65.5 ^cd^	24.4 ^c^	7.00 ^ab^	0.73 ^b^	0.84 ^ab^	1.54 ^a^	3.22 ^cd^	5.75 ^bc^	27.8 ^b^	8.69 ^bc^	84.2 ^c^
*Pelvetia canaliculata*	Spring	1.08	65.9	21.1	7.05	0.46	1.06	4.37	3.13	7.28	4.91	4.54	70.1
	Autumn	1.10	64.6	19.4	10.1	0.53	1.01	4.34	3.40	5.58	6.10	5.55	69.3
	Average	1.09 ^a^	65.3 ^cd^	20.3 ^b^	8.60 ^b c^	0.50 ^a^	1.04 ^b^	4.36 ^c^	3.27 ^cde^	6.43 ^c^	5.50 ^a^	5.05 ^a^	69.7 ^b^
*Saccharina latissima*	Spring	3.56	74.9	16.3	4.85	0.93	1.30	1.74	4.61	9.86	24.6	6.91	92.8
	Autumn	4.62	52.7	35.4	9.22	0.78	0.65	1.29	1.49	0.51	47.1	10.2	85.2
	Average	4.09 ^d^	63.8 ^b c^	25.8 ^cd^	7.00 ^ab^	0.86 ^c^	0.98 ^b^	1.52 ^a^	3.05 ^c^	5.18 ^ab^	35.9 ^b^	8.78 ^b^	89.0 ^de^
Red seaweeds													
*Mastocarpus stellatus*	Spring	2.64	67.6	15.8	9.30	1.38	2.81	3.05	4.28	15.1	18.0	6.82	88.3
	Autumn	1.44	68.3	14.3	9.66	1.49	2.99	3.22	4.76	13.5	11.1	7.71	88.8
	Average	2.04 ^ab^	67.9 ^e^	15.1 ^a^	9.50 ^c^	1.44 ^e^	2.90 ^c^	3.14 ^b^	4.52 ^f^	14.3 ^e^	14.6 ^a^	7.16 ^a^	88.6 ^d^
*Palmaria palmata*	Spring	7.18	61.1	26.4	6.65	1.33	1.71	2.76	2.31	22.1	64.6	9.00	94.9
	Autumn	6.56	55.8	33.7	7.89	0.70	0.55	1.35	1.67	0.78	60.2	9.18	88.7
	Average	6.87 ^e^	58.5 ^a^	30.1 ^e^	7.30 ^ab^	1.02 ^d^	1.13 ^b^	2.06 ^a^	1.99 ^a^	11.4 ^d^	62.4 ^c^	9.08 ^b c^	91.8 ^e^
*Porphyra sp.*	Spring	3.28	66.4	16.1	8.33	2.42	3.66	3.17	4.18	39.9	34.3	10.5	82.7
	Autumn	3.00	61.0	19.9	9.28	2.62	4.00	3.23	3.09	37.7	39.3	13.1	79.9
	Average	3.14 ^b c^	63.7 ^b c^	17.9 ^b^	8.80 ^b c^	2.52 ^g^	3.83 ^d^	3.20 ^b^	3.64 ^e^	38.8 ^g^	36.8 ^b^	11.7 ^cd^	81.3 ^c^
Green seaweeds													
*Cladophorarupestris*	Spring	2.38	69.0	16.0	4.97	2.07	3.66	4.25	4.32	29.8	31.9	13.4	63.9
	Autumn	3.00	63.3	24.4	6.59	1.40	1.85	2.43	2.60	19.5	35.9	12.0	62.0
	Average	2.70 ^b c^	66.2 ^de^	20.2 ^b^	5.80 ^a^	1.74 ^f^	2.76 ^c^	3.34 ^b^	3.46 ^de^	24.7 ^f^	33.9 ^b^	12.6 ^e^	63.0 ^a^
													
*p* value													
Species		<0.001	<0.001	<0.001	0.013	<0.001	<0.001	<0.001	<0.001	<0.001	<0.001	0.001	<0.001
Season		0.821	<0.001	<0.001	<0.001	<0.001	<0.001	0.021	<0.001	<0.001	<0.001	0.083	<0.001
Species x season		0.609	<0.001	<0.001	0.104	<0.001	<0.001	0.066	<0.001	<0.001	0.267	0.312	<0.001
SEM		0.154	0.310	0.330	0.249	0.015	0.041	0.086	0.047	0.164	1.588	0.394	0.355
Feeds													
Oat hay		3.84	68.6	19.5	8.10	0.75	0.86	2.13	3.52	6.09	28.2	7.34	64.0
Barley straw		2.68	69.3	20.4	7.03	0.70	0.91	1.66	3.40	2.97	21.7	8.10	38.0
Commercial concentrate		5.48	57.5	25.7	1.10	1.44	1.87	2.53	2.24	12.2	56.4	10.3	86.8

^a–g^ For each parameter, the average values for each seaweed not sharing the same superscript differ (*p* < 0.05).

**Table 5:** Chemical composition (g/kg dry matter (DM) unless otherwise stated) of diets containing 50% of oat hay and 50% of concentrate either including no seaweeds (control) or different seaweed species harvested in spring and autumn in northern Norway.

Seaweed Species	Harvesting Season	Concentrate	Dry Matter (g/kg Fresh Matter)	Ash	Nitrogen	Ether Extract	Total Extractable Polyphenols
-	-	Control	924	68.6	17.9	17.7	5.92
*Alaria esculenta*	Spring	AS	902	86.4	19.8	16.6	5.73
	Autumn	AA	901	72.1	18.4	17.8	6.29
*Laminaria digitata*	Spring	LS	910	81.9	19.4	13.8	5.64
	Autumn	LA	911	109	17.6	14.4	5.98
*Pelvetia* *canaliculata*	Spring	PS	904	77.9	18.6	15.8	5.69
	Autumn	PA	903	85.9	17.6	15.8	6.10
*Saccharina* *latissima*	Spring	SS	905	97.9	19.5	15.9	4.82
	Autumn	SA	905	79.5	17.8	16.9	6.58
*Mastocarpus* *stellatus*	Spring	MS	904	80.9	19.4	14.8	4.75
	Autumn	MA	905	79.7	18.1	15.5	5.83
*Palmaria* *palmata*	Spring	PPS	907	76.7	18.7	15.5	4.99
	Autumn	PPA	908	80.5	17.6	15.8	4.79
*Porphyra sp.*	Spring	POS	902	70.6	17.8	15.9	5.65
	Autumn	POA	901	64.1	18.1	15.8	5.96
*Cladophora* *rupestris*	Spring	CS	902	60.2	18.7	16.4	4.99
	Autumn	CA	900	70.8	17.8	16.6	5.62

**Table 6:** Parameters of gas production kinetics (A, c and AGPR) after 144 h of in vitro incubation of diets containing 50% of oat hay and 50% of concentrate either including no seaweeds (control) or different seaweed species harvested in spring and autumn in northern Norway ^1^.

Seaweed Species	Harvesting Season	Concentrate	A (ml)	c (h^−1^)	AGPR (ml/h)
-	-	Control	138 ^a^	0.050 ^b^	4.98 ^b^
*Alaria* *esculenta*	Spring	AS	134 ^a^	0.044 ^a^	4.30 ^a^
	Autumn	AA	138 ^a^	0.043 ^a^	4.28 ^a^
*Laminaria digitata*	Spring	LS	131 ^a^	0.040 ^a^	3.78 ^a^
	Autumn	LA	133 ^a^	0.042 ^a^	4.03 ^a^
*Pelvetia* *canaliculata*	Spring	PS	136 ^a^	0.041 ^a^	4.02 ^a^
	Autumn	PA	129 ^a^	0.041 ^a^	3.82 ^a^
*Saccharina* *latissima*	Spring	SS	133 ^a^	0.043 ^a^	4.13 ^a^
	Autumn	SA	137 ^a^	0.043 ^a^	4.25 ^a^
*Mastocarpus* *stellatus*	Spring	MS	135 ^a^	0.044 ^a^	4.28 ^a^
	Autumn	MA	131 ^a^	0.043 ^a^	4.03 ^a^
*Palmaria* *palmata*	Spring	PPS	135 ^a^	0.045 ^a^	4.38 ^a^
	Autumn	PPA	145 ^b^	0.047 ^ab^	4.92 ^b^
*Porphyra sp.*	Spring	POS	147 ^b^	0.041 ^a^	4.35 ^a^
	Autumn	POA	148 ^b^	0.042 ^a^	4.48 ^a^
*Cladophora* *rupestris*	Spring	CS	146 ^b^	0.041 ^a^	4.35 ^a^
	Autumn	CA	149 ^b^	0.040 ^a^	4.30 ^a^
*p* value			<0.001	0.033	0.215
SEM			0.56	0.0014	0.098

^a-b^ For each parameter, the mean values for each diet not sharing the same superscript differ (*p* < 0.05). ^1^A: asymptotic gas production; c: rate of gas production; AGPR: average gas production rate. The values of *lag* were 0 for all samples. Data are expressed per 0.5 g DM fermented.

**Table 7:** Fermentation parameters after 24 h of in vitro incubation of diets containing 50% of oat hay and 50% of concentrate either including no seaweeds (control) or different seaweed species harvested in spring and autumn in northern Norway ^1^.

Seaweed Species	Harvesting Season	Concentrate	VFA (mmol/g DM)	Molar Proportions (mol/100 mol)						Acetate/Propionate (mol/mol)	NH_3_-N (mg/100 mL)	CH_4_ (mL/g DM)	CH_4_/VFA (mL/mmol)
				Acetate	Propionate	Butyrate	Isobutyrate	Isovalerate	Valerate				
-	-	Control	5.06	62.8 ^a^	21.2 ^b^	11.9 ^b^	0.98	1.20	1.79	2.96 ^a^	10.2	64.6 ^ab^	12.8
*Alaria esculenta*	Spring	AS	4.88	65.3^b^	19.3 ^a^	11.3 ^a^	0.99	1.38	1.73	3.39^b^	9.88	65.4 ^b^	13.4
	Autumn	AA	5.02	63.8 ^a^	22.5 ^c^	10.4 ^a^	0.88	1.03	1.49	2.84 ^a^	7.09	63.2 ^a b^	12.4
*Laminaria digitata*	Spring	LS	4.86	65.8 ^b^	19.8 ^a^	10.5 ^a^	0.97	1.32	1.65	3.33 ^b^	9.84	64.0 ^a b^	13.2
	Autumn	LA	4.86	64.6 ^b^	21.8 ^a^	9.93 ^a^	0.91	1.14	1.63	2.96 ^a^	7.57	61.9 ^a^	12.7
*Pelvetia canaliculata*	Spring	PS	4.80	64.8 ^b^	19.4 ^a^	11.9 ^b^	0.95	1.31	1.65	3.34 ^b^	10.1	63.6 ^a b^	13.2
	Autumn	PA	5.06	65.0 ^b^	19.7 ^a^	11.0 ^b^	1.04	1.36	1.81	3.30 ^b^	8.10	66.6 ^b^	13.2
*Saccharina latissima*	Spring	SS	4.86	65.2 ^b^	20.3 ^a^	10.6 ^a^	0.98	1.31	1.65	3.22 ^b^	9.49	63.1 ^a b^	13.0
	Autumn	SA	5.22	63.6 ^a^	21.8 ^a^	10.6 ^a^	0.99	1.30	1.68	2.91 ^a^	7.83	65.0 ^b^	12.4
*Mastocarpus stellatus*	Spring	MS	4.90	64.6 ^b^	19.4 ^a^	12.0 ^b^	1.02	1.35	1.58	3.32 ^b^	10.3	65.5 ^b^	13.4
	Autumn	MA	4.66	64.7 ^b^	19.5 ^a^	11.9 ^b^	0.99	1.32	1.64	3.31 ^b^	9.51	61.6 ^a^	13.2
*Palmaria palmata*	Spring	PPS	5.26	64.3 ^b^	19.7 ^a^	12.0 ^b^	1.03	1.31	1.64	3.26 ^b^	10.4	68.4 ^b^	13.0
	Autumn	PPA	5.26	63.8 ^a^	20.6 ^a^	11.9 ^b^	0.96	1.22	1.51	3.10 ^ab^	8.87	67.7 ^b^	12.9
*Porphyra sp.*	Spring	POS	5.16	63.6 ^a^	19.9 ^a^	12.6 ^c^	0.95	1.30	1.62	3.19 ^b^	9.34	67.2 ^b^	13.0
	Autumn	POA	5.18	63.8 ^a^	19.5 ^a^	12.6 ^c^	1.03	1.36	1.66	3.27 ^b^	8.61	66.1 ^b^	12.8
*Cladophora rupestris*	Spring	CS	5.00	63.6 ^a^	20.0 ^a^	12.5 ^c^	0.95	1.26	1.67	3.19 ^b^	8.44	65.2 ^b^	13.0
	Autumn	CA	5.24	63.8 ^a^	19.6 ^a^	12.8 ^c^	0.98	1.30	1.53	3.26 ^b^	8.95	67.5 ^b^	13.2
*p*-value			0.152	<0.001	0.008	<0.001	0.767	0.861	0.636	0.033	0.960	0.049	0.569
SEM			0.042	0.088	0.129	0.063	0.011	0.025	0.023	0.023	0.349	1.59	0.52

^a–c^ For each parameter, the mean values for each diet not sharing the same superscript differ (*p* < 0.05).
